# Functional Disability After Ischemic Stroke: A Community-Based Cross-Sectional Study in Shanghai, China

**DOI:** 10.3389/fneur.2021.649088

**Published:** 2021-08-26

**Authors:** Ying-Ye Yao, Zi-Jun Wei, Yue-Chan Zhang, Xiang Li, Liu Gong, Jia-Wei Zhou, Yu Wang, Yun-Yun Zhang, Rui-Ping Wang

**Affiliations:** ^1^Department of Neurology, Yueyang Hospital of Integrated Traditional Chinese and Western Medicine Affiliated to Shanghai University of Traditional Chinese Medicine, Shanghai, China; ^2^Department of Neurology, Shanghai Baoshan Integrated Traditional Chinese and Western Medicine Hospital, Shanghai, China; ^3^Clinical Research Center, Shanghai Skin Diseases Hospital, Tongji University, Shanghai, China

**Keywords:** functional status, ischemic stroke, living dependence, community, cross-sectional survey

## Abstract

**Objective:** This study aimed to understand the demographics, functional disabilities, cognitive impairment, and depressive mood among stroke patients and to explore the correlation between functional disability and the other health conditions so as to provide some data for community rehabilitation among stroke patients.

**Methods:** A cross-sectional study was conducted to investigate the functional status of ischemic stroke patients with stroke history between 1 month and 2 years by applying the modified Rankin Scale (mRS). Data were collected during October 2016 and January 2017 from 11 communities in two districts of Shanghai, China. We used face-to-face questionnaire interviews to collect information on sociodemographics, vascular risks associated with stroke, cognitive function [Mini-Mental State Examination (MMSE)], and depression [Patient Health Questionnaire-9 (PHQ-9)]; and we applied SPSS 24.0 for data analysis.

**Results:** In this study, 305 patients with ischemic stroke were finally recruited, including 189 (61.97%) men, with an average age of 67 years. According to the mRS score, ischemic stroke patients were divided into patients without symptoms (controls, mRS = 0), patients without obvious disability (mRS = 1), and patients with mild to severe disability (mRS = 2–5). Ischemic stroke patients with different mRS levels demonstrated significant differences in age, tobacco smoke exposure, previous stroke history, cognitive function, and depression status. Compared with patients without symptoms (mRS = 0), patients with mRS = 1 had a lower MMSE score [odds ratio (OR): 0.48, 95% confidence interval (CI): 0.26–0.90]; and patients with mRS = 2–5 had a lower MMSE score [OR = 0.16, 95% CI: 0.08–0.33], had a higher PHQ-9 score [OR = 5.36, 95% CI: 2.19–13.11], and were more likely to have previous stroke history [OR = 2.18, 95% CI: 1.01–4.79].

**Conclusion:** Lower degrees of functional independence are related to cognitive impairment, as well as the previous stroke history and depression status.

## Introduction

Stroke is one of the worldwide leading causes of death and disability (1). In China, according to the China National Stroke Screening Survey, the incidence of primary stroke in adults aged 40–74 years increased from 189/100,000 in 2002 to 379/100,000 in 2013, although the stroke-specific mortality remained at approximately 124/100,000 in both 2002 and 2013 (2). Previous studies demonstrated high global incidence, recurrence, and mortality rates of stroke, as well as high prevalence of disability due to stroke among stroke survivors. The American Heart Association reported that nearly 75% of stroke victims have dysfunction and 15–30% of stroke survivors have severe disability (3). In China, the national 2010 survey demonstrated a 45% prevalence of disability among stroke survivors (4). Based on all the above information, the functional disability among ischemic stroke (IS) patients warrants more attention.

Previous studies on the health status of stroke patients mainly focused on complicated accompanying diseases and physical disabilities (4–9), while studies on functional disability in China are limited. Functional disability cannot be isolated from other health indicators, such as sociodemographic differences, vascular risks factors, cognitive function [Mini-Mental State Examination (MMSE)], and depression [Patient Health Questionnaire-9 (PHQ-9)]. Snaphaan reported dynamic changes in cognitive impairment after stroke: the prevalence of post-stroke memory dysfunction declined almost 50% from 3 months to 1 year post-stroke (10). Further, Ferri indicated that the proportion of stroke survivors in Latin America requiring medical care for cognitive impairment ranged from 20 to 39%, with a higher proportion in both urban and rural China (11). Ojagbemi found a 31% pooled frequency of clinically diagnosed post-stroke depression and that post-stroke depression was significantly associated with cognitive impairment and physical disability (12). This research focused on dysfunction and analyzed the link between dysfunction and other health conditions, aimed to provide some data for community rehabilitation of stroke patients.

## Materials and Methods

### Study Population

We implemented this cross-sectional study in 11 communities in two districts of Shanghai, China, between October 2016 and January 2017. The 11 communities covered seven communities in the Jiading District and four in the Minhang District. We recruited IS patients in the community health service centers of all 11 communities. In this study, the Ethics Committee of Yueyang Hospital of Integrated Traditional Chinese and Western Medicine Affiliated to Shanghai University of Traditional Chinese Medicine (2016 Ethical Review No. 036) approved the study. Written informed consent was provided by each participant before the questionnaire interview.

In this study, IS was defined as blood clot- or plaque fragment-related blockage of a blood vessel supplying the brain. Qualifying patients with novel IS and disease duration between 1 month and 2 years were recruited.

Inclusion criteria were as follows: (i) local residents, aged 40–80 years; (ii) IS diagnosed by physicians in accordance with the 10th edition of the International Classification of Diseases (ICD-10) codes 163–169 and confirmed by head magnetic resonance imaging (MRI) with the event of stroke occurring between 1 and 24 months (≥1 and ≤24 months). Exclusion criteria included (i) stroke patients with serious chronic diseases and malignant tumors; and (ii) patients with mental illnesses who could not cooperate with the investigation.

### Sample Size Estimation

In this study, we use the proportion of patients with mild to severe disability (modified Rankin Scale (mRS) value between 2 and 5) as the index to estimate the sample size. A previous study (4) demonstrated that the proportion of patients with mild to severe disability was about 45%, by applying the following sample size calculation for cross-sectional studies:

$$\begin{array}{l} n=\frac{Z_{a/2}^{2}\times p\left(1-p\right)}{\delta^{2}} \end{array}$$

where the significance level *α* is 0.05, and the error *δ* is 13% of the proportion, resulting in a sample size of 277. Considering a 10% refusal rate, ≥305 IS patients should be recruited for this study.

### Group Classification

**Figure 1:**
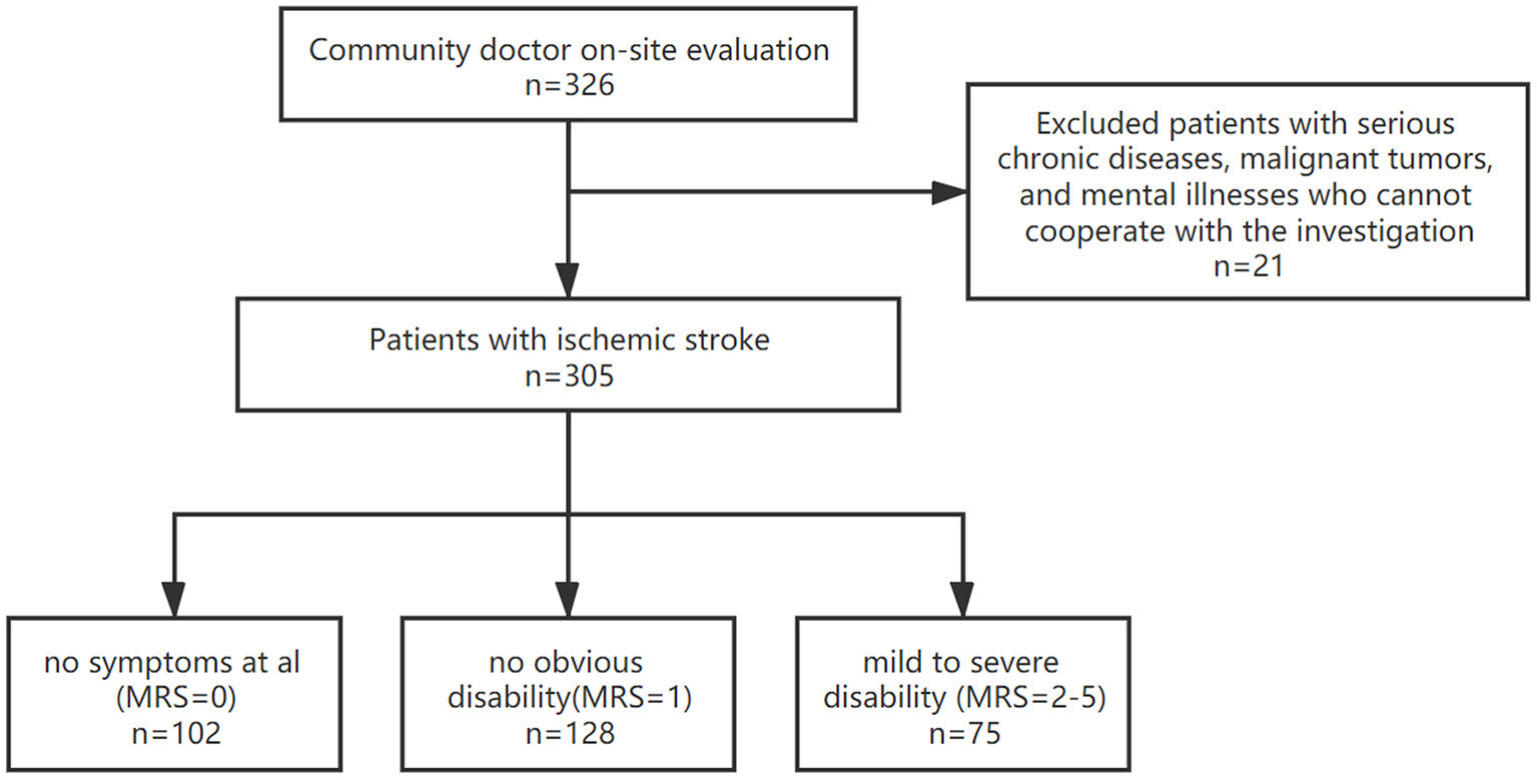
Flow chart of the cross-sectional study among ischemic stroke patients.

In this study, 305 patients with IS were recruited. We then divided all patients into three groups according to their functional status. Functional status was assessed using the mRS and divided into the following criteria: favorable (mRS of 0 or 1) or unfavorable outcomes (mRS 2–5) (13). The first group included patients without clinical symptoms and an mRS = 0; the second, patients with no obvious disability and mRS = 1; and the third, patients with mild to severe disability and mRS = 2–5 (Figure 1).

### Data Collection

In this study, all participants were initially screened by a face-to-face interview; then information on sociodemographic features, stroke history, vascular risks associated with stroke, functional disabilities, cognition, and depression was collected via a structured questionnaire by pre-trained investigators.

Part A included sociodemographic information: gender, age, ethnicity, marital status, profession, level of education, and stroke duration.

Part B included information on stroke associated vascular risks. We first extracted the patients' detailed clinical history from the health information system (HIS) in all hospitals, and then we investigated their medical records for vascular risk factors according to the Essen Stroke Risk Score (ESRS), including hypertension, diabetes, peripheral arterial disease, stroke history, smoking status, transient ischemic attack (TIA) history, and myocardial infarction history; stroke patients with higher ESRS scores are at a higher risk of stroke recurrence (14).

Part C corresponded to functional information. The mRS was used to demonstrate functional disability; it has six grades to rank the degree of disability. Grade 0 indicates no symptoms at all; grade 1 indicates no significant disability despite of symptoms (i.e., ability to perform all usual duties and activities); and grade 2–5 indicates slight to severe disability (i.e., patients cannot perform all previous activities or their condition worsens). mRS was characterized according to the criteria: favorable outcome (mRS of 0 or 1) or unfavorable outcome (mRS 2–5) (13). The assessment was implemented by asking patients questions on their daily activities, including outdoor ones (15).

Part D included cognitive information. The MMSE was used for cognitive screening. The maximum total score allotted was 30. The MMSE contains two sections. The first requires a vocal response, which measures orientation, memory, and attention. The second was used to verify patients' ability to name and follow verbal and written commands. A score between 27 and 30 indicated a normal range, and a score <27 demonstrated cognitive dysfunction. The severity of dementia could also be evaluated as follows: scores ≥21 were treated as mild MMSE, scores between 10 and 20 as moderate MMSE, and scores ≤9 as severe MMSE. MMSE evaluation operators were instructed to make patients comfortable and to avoid emphasizing difficult situations for the patients (16).

Part E included information on depression. The PHQ-9 was used to screen for depression. PHQ-9 is a self-administered diagnostic instrument for common mental disorders. The PHQ-9 scores each of nine criteria with scales between “0” (not at all) and “3” (nearly every day). As a severity measure, the PHQ-9 score ranges from 0 to 27. A score 0–4 indicates no depression; and scores 5–9, 10–14, 15–19, and 20–27 indicate mild to severe depression. The reported mild depression cutoff in the PHQ-9 is 5 (17, 18). Prior to the final score, the evaluation operator ruled out physical causes of depression, normal bereavement, and a history of manic episodes (19).

### Statistical Analysis

We applied SPSS version 24.0 for data analysis. We described the data by using frequency counts and proportions (percentage) for qualitative variables, and mean and standard deviation (SD) or median and interquartile range (IQR) for quantitative variables. We applied the chi-square test to examine differences of demographic features, stroke history, vascular risks associated with stroke, function, cognition, and depression among stroke patients with different mRS scores. We applied logistic regression to calculate odds ratios (ORs) and 95% confidence intervals (95% CIs) to explore the association between stroke patients with different mRS scores and other health conditions. Patients with mRS = 0 were used as reference, and age (41–60 years = 1; and 61–80 years = 2), smoking status (yes = 1 and no = 0), stroke history (yes = 1 and no = 0), MMSE score (0–26 points = 1; and 27–30 points = 2), and PHQ-9 scores (0–4 points = 1; and 5–27 points = 2) were included as independent variables in the model. A *p*-value <0.05 (two-tailed) was considered statistically significant.

## Results

### Demographic Features

In this study, 305 patients with IS were finally included for data analysis. Among them, 189 (61.97%) were male, with an average age of 67 years (SD 7.84); 77.05% were between 61 and 80 years old, and 303 (99.34%) had Han ethnicity. In this study, 279 (91.48%) stroke patients were married, 270 (88.52%) were retired, and 150 (49.18%) had an education of middle school or higher level, and 248 (81.31%) stroke events occurred in patients with disease duration >6 months (Table 1).

**Table 1:** The sociodemographic features of ischemic stroke patients.

**Social demographic features**	**Patients with ischemic stroke**
Sex, n (%)	
Male	189 (61.97)
Female	116 (38.03)
Age (years), n (%)	
41~60	70 (22.95)
61~80	235 (77.05)
Average age (years), (mean, SD)	67 ± 7.84
Ethnicity, n (%)	
Han nationality	303 (99.34)
Non-Han nationality	2 (0.66)
Marital situation, n (%)	
Married	279 (91.48)
Single	5 (1.64)
Widowed	18 (5.90)
Divorce	3 (0.98)
Profession, n (%)	
Serving	35 (11.48)
Retire	270 (88.52)
Level of education, n (%)	
Illiterate	32 (10.49)
Elementary school	103 (33.77)
Middle school	150 (49.18)
College degree and above	20 (6.56)
Stroke course, n (%)	
≥1 and <6 months	57 (18.69)
≥6 months and ≤2 years	248 (81.31)

### Vascular Risk Factors

Table 2 indicates the most common vascular risk factors in patients with IS, including hypertension (78.03%), diabetes (28.20%), tobacco smoking (26.56%), and a history of previous stroke (22.95%) (Table 2).

**Table 2:** Vascular risk factors of ischemic stroke patients (*n* = 350).

**Vascular risk factors**	**Ischemic stroke patients**
Hypertension	238 (78.03)
Diabetes	86 (28.20)
Tobacco smoking	81 (26.56)
Previous stroke history	70 (22.95)
Previous TIA history	12 (3.93)
Previous myocardial infarction history	7 (2.30)
Peripheral arterial disease	7 (2.30)

*TIA, transient ischemic attack*.

### Health Status

In this study, mRS, MMSE, and PHQ-9 scores were used to demonstrate the functional status, intelligence, and depressive moods among stroke patients, respectively (Table 3). We found that 66.56% of stroke patients had various degrees of sequelae (mRS score from 1 to 5), while 38.69% of patients had different levels of cognitive impairment (MMSE score ≤ 26), and 19.02% had depression.

**Table 3:** Health status of ischemic stroke patients (*n* = 350).

**Functional status**	**Ischemic stroke patients**
Dependence on daily activities (mRS)	
0 (no symptoms at all)	102 (33.44)
1 (no obvious disability)	128 (41.97)
2 (mild disability)	24 (7.87)
3 (moderate disability)	29 (9.51)
4 (moderate to severe disability)	15 (4.92)
5 (severe disability)	7 (2.30)
Intelligence status check (MMSE)	
Normal (27–30 points)	187 (61.31)
Mild (21–26 points)	73 (23.93)
Moderate (10–20 points)	34 (11.15)
Severe (0–9 points)	11 (3.61)
Self-assessment of depression (PHQ-9)	
No depression (0–4 points)	247 (80.98)
Mild depression (5–9 points)	40 (13.11)
Moderate depression (10–14 points)	12 (3.93)
Moderate to severe depression (15–19 points)	4 (1.31)
Severe depression (20–27 points)	2 (0.66)

*mRS, modified Rankin Scale; MMSE, Mini-Mental State Examination; PHQ-9, Patient Health Questionnaire-9*.

### Functional Status

Table 4 shows that a statistically higher proportion of older stroke patients, tobacco smokers, and patients with stroke history and lower MMSE and PHQ-9 scores had an mRS score between 2 and 5 (*p* < 0.05) (Table 4).

**Table 4:** Comparison between basic data, vascular risk factors and health status among ischemic stroke patients (*n* = 350).

**Features**		**mRS (n, %)**			**Total** **(*n* = 350)**	**χ** ^**2**^	***p***
		**0 (*n* = 102)** **(no symptoms)**	**1 (*n* = 128)** **(no obvious)**	**2–5 (*n* = 75)** **(mild to severe)**			
Gender	Male	61 (59.80)	79 (61.72)	49 (65.33)	189 (61.97)	0.57	0.75
	Female	41 (40.20)	49 (38.28)	26 (34.67)	116 (38.03)		
Age (years)	41–60	32 (31.37)	27 (21.09)	11 (14.67)	70 (22.95)	7.25	0.03^*^
	61–80	70 (68.63)	101 (78.91)	64 (85.33)	235 (77.05)		
Education	Elementary/illiterate	41 (40.20)	55 (42.97)	39 (52.00)	135 (44.26)	2.59	0.27
	Middle school and above	61 (59.80)	73 (57.03)	36 (48.00)	170 (55.74)		
Stroke course	≥1 and <6 months	15 (14.71)	29 (22.66)	13 (17.33)	57 (18.69)	2.48	0.29
	≥6 months and ≤2 years	87 (85.29)	99 (77.34)	62 (82.67)	248 (81.31)		
Hypertension	Yes	20 (19.61)	24 (18.75)	23 (30.67)	67 (21.97)	4.42	0.11
	No	82 (80.39)	104 (81.25)	52 (69.33)	238 (78.03)		
Diabetes	Yes	69 (67.65)	95 (74.22)	55 (73.33)	219 (71.80)	1.33	0.52
	No	33 (32.35)	33 (25.78)	20 (26.67)	86 (28.20)		
Smoking	Yes	66 (64.71)	101 (78.91)	57 (76.00)	224 (73.44)	6.20	0.05
	No	36 (35.29)	27 (21.09)	18 (24.00)	81 (26.56)		
Stroke history	Yes	85 (83.33)	105 (82.03)	45 (60.00)	235 (77.05)	16.40	<0.01^**^
	No	17 (16.67)	23 (17.97)	30 (40.00)	70 (22.95)		
MMSE	Normal (27–30 points)	82 (80.39)	81 (63.28)	24 (32.00)	187 (61.31)	43.03	<0.01^**^
	Mild to severe (0–26 points)	20 (19.61)	47 (36.72)	51 (68.00)	118 (38.69)		
PHQ-9	Normal (0–4 points)	93 (91.18)	109 (85.16)	45 (60.00)	247 (80.98)	29.77	<0.01^**^
	Mild to severe (5–27 points)	9 (8.82)	19 (14.84)	30 (40.00)	58 (19.02)		

*mRS, modified Rankin Scale; MMSE, Mini-Mental State Examination; PHQ-9, Patient Health Questionnaire-9*.

* *p < 0.05*.

** *p < 0.01*.

### Association of Functional Status With Other Health Conditions

Table 5 shows that in comparison with IS patients with an mRS = 0, patients with an mRS = 1 had a significantly lower MMSE score (OR: 0.48, 95% CI: 0.26–0.90). Further, in comparison with IS patients with an mRS = 0, patients with an mRS = 2–5 had a significantly lower MMSE score (OR: 0.16, 95% CI: 0.08–0.33). Meanwhile, a higher proportion of stroke patients with an mRS = 2–5 had a previous stroke history (OR: 2.18, 95% CI: 1.01–4.79) and higher PHQ-9 scores (OR: 5.36, 95% CI: 2.19–13.11).

**Table 5:** Multivariable logistic regression analysis among patients with different mRS groupings.

**mRS = 1 vs. 0**	**Regression coefficient**	**Standard error**	**Wald χ** ^**2**^	**OR**	**95% CI**
Age	0.38	0.32	1.43	1.46	0.79~2.70
Smoking	−0.59	0.31	3.58	0.56	0.30~1.02
Previous stroke history	−0.01	0.37	0.001	0.99	0.48~2.03
MMSE	−0.73	0.32	5.29	**0.48**	**0.26~0.90**
PHQ-9	0.53	0.44	1.47	1.70	0.72~4.04
Intercept	0.40	0.99	0.17	1.50	0.22~10.32
**mRS = 2–5 vs. 0**	**Regression coefficient**	**Standard error**	**Wald χ** ^**2**^	**OR**	**95% CI**
Age	0.72	0.44	2.65	2.06	0.86~4.91
Smoking	−0.22	0.39	0.32	0.80	0.37~1.73
Previous stroke history	0.78	0.40	3.80	**2.18**	**1.01~4.79**
MMSE	−1.85	0.37	24.86	**0.16**	**0.08~0.33**
PHQ-9	1.68	0.46	13.55	**5.36**	**2.19~13.11**
Intercept	−0.86	1.21	0.50	0.43	0.04~4.55

*McFadden R^2^: 0.12*

*CI, confidence interval; mRS, modified Rankin Scale; MMSE, Mini-Mental State Examination; PHQ-9, Patient Health Questionnaire-9. The bold value means the correlation was statistically significant*.

## Discussion

In this study, we found that 66.56% of IS patients had sequelae symptoms and 24.59% varying degrees of disability (mRS ≥ 2). Yang et al. reported a 5-year disability rate (mRS ≥ 2) of 45% among 893 patients with IS in China (4). Ong et al. (20) investigated 4,278 patients with IS in Taiwan and indicated a good prognosis rate of 41.2% (1,761/4,278) (mRS ≤ 2) at hospital discharge, which increased to 56.1% (1,813/3,231) after 6 months. Ruiz-Sandoval et al. (21) investigated 1,246 patients in Mexico and found good prognosis (mRS ≤ 2) in 34% of IS patients during the first month after stroke, while 41% showed a good prognosis 1 year after stroke onset. In our study, the proportion of IS patients with good prognosis was higher than that of the abovementioned studies; this is because the stroke patients in our study were recruited in the community, thus excluding those still in the rehabilitation hospital, or because the disease duration of IS patients in this study was restricted within 2 years.

Cognitive level was negatively correlated with functional disability. In the present study, 38.69% of stroke patients showed cognitive impairment (MMSE ≤ 26). Arba et al. (22) found that 781 (34%) patients at 1 year and 391 (30%) patients at 3 years after stroke had an MMSE ≤ 26. Pendlebury et al. (23) found that the overall prevalence of dementia among 2,305 stroke patients in Oxford (England) after 1 year was 46.8%. However, to determine the causal association between cognition decline and functional disability in stroke patients, a longer follow-up period is required. The decrease of physical function and cognitive function among stroke patients was explained by its common simultaneous manifestation with stroke. Vaughan et al. (24) followed up 59 female stroke survivors aged between 65 and 79 years during 8.12 (2.30) years, and they found that higher physical function was protective against the global post-IS cognitive decline. Abzhandadze et al. (25) observed 305 stroke patients and indicated that the Montreal Cognitive Assessment (MoCA) cognitive assessment tool within 36–48 h of stroke could predict functional dependence at 3 months. Li et al. (26) interviewed 185 stroke patients during the acute phase as well as 1, 3, and 6 months after stroke onset and found that patients with cognitive impairment had higher risks of disability than those with normal cognitive conditions. Our research results and many literature records suggest that functional disability is related to cognitive decline.

Our analysis indicated that depression was a slight to severe disability among IS patients, compared with IS patients without symptoms. Particularly, 19.02% of patients suffered from depression (PHQ-9 ≥ 5). Wu (27) systematically analyzed six studies and 4,648 stroke patients and demonstrated a prevalence of post-stroke depression from 15.9 to 40.5%. Arba et al. (28) enrolled 2,160 patients with IS and found that 416 (19.3%) patients had depression symptoms 1 year after stroke. Jørgensen et al. (29) studied 157,243 stroke patients in Denmark and found a higher prevalence of depression in stroke patients in the first 3 months of hospitalization, which decreased in the second year. In this study, we recruited stroke patients who suffered their last stroke event between 1 and 24 months. The proportion of IS patients with depression in our study was in accordance with that of previous ones, suggesting a mutual detrimental relationship between body function and depression. Dušica et al. (30) found that cognitive decline and depression were negative determinants of rehabilitation outcomes in patients with IS. Oni et al. (31) observed 70 stroke survivors and found a significantly higher severity of disability in patients with post-stroke depression than without. Vojtikiv-Samoilovska et al. (32) studied the prevalence and risk factors of depression among 100 stroke patients and found that post-stroke depression was related to the Activity of Daily Living. These results are consistent with our findings, indicating that more attention should to be given to the emotional regulation of IS patients.

Another demographic feature associated with the worsening functional status of IS patients was stroke history. This result may be due to the accumulation of multiple stroke sequelae. Various reports demonstrated that previous stroke history is associated with poor outcomes (33–35). And other studies also indicated that previous stroke history is an indicator of poor physical function among IS patients (36, 37).

Although tobacco smoke was an intervening factor, it was difficult to clarify the positive or negative relationship between smoking and functional status of IS patients. Campos et al. (38) indicated that the acute nicotine effects improved the levels of cognitive performance in smokers, while cognitive disruption resulted from nicotine abstinence. Markidan et al. (39) identified a significantly increased risk of IS in smokers (risk ratio: 1.88), with a dose-response relationship. In any event, as tobacco smoking is a recognized risk factor for stroke, smoking cessation is necessary to prevent stroke.

Some research demonstrated that stroke-associated diseases affect the recovery of stroke patients. Simić-Panić et al. (40) found that European stroke patients with hypertension, diabetes, myocardial infarction, and atrial fibrillation had relatively poor rehabilitation after stroke and a high dependence for activities of daily life. Soriano-Reixach et al. (41) conducted a follow-up study of IS patients and found a significantly higher 5-year disability level and mortality rate among stroke patients with diabetes.

This study has some limitations. First, mRS, MMSE, and PHQ-9 were self-reported, which might lead to overreporting and an equal proportion of misclassified stroke patients from low to higher scores, leading to a potential risk of OR underestimation. Second, according to previous research, the recommended cutoff mRS score is 2 and that of PHQ-9 is 10 (42, 43), while the Chinese version of PHQ-9 has a cutoff of 7 (44). In our study, reducing both cutoff scores (mRS to 1 and PHQ-9 to 5) reduced their specificity. The participants in our study came from a community, so their health status was better than that of participants from a hospital or rehabilitation center. Considering the characteristics of the participants in our study, our cutoff scores may not be appropriate, and the functional disability and depression were overestimated. Third, the participants had a long course of disease, and the sample size is insufficient for more detailed subgroup analyses. In the future, a larger sample size and a more detailed subgroup analysis are needed.

## Conclusion

IS patients without obvious disability levels (mRS score 1) are associated with lower MMSE, while those with mild to severe disability levels (mRS score 2–5) are associated with a previous stroke history, lower MMSE, and higher PHQ-9. We recommend specific rehabilitation and care programs for the various basic conditions of stroke patients. Simultaneously, during rehabilitation, attention should be paid to dysfunctional stroke patients with cognitive impairment, previous stroke history, and depression.

## Data Availability Statement

The raw data supporting the conclusions of this article will be made available by the authors, without undue reservation.

## Ethics Statement

The studies involving human participants were reviewed and approved by the Ethics Committee of Yueyang Hospital of Integrated Traditional Chinese and Western Medicine Affiliated to Shanghai University of Traditional Chinese Medicine. The patients/participants provided their written informed consent to participate in this study.

## Author Contributions

Y-YZ and LG participated in the study design. LG, J-WZ, and YW conducted the study and drafted the paper. Y-YY, Z-JW, Y-CZ, and XL participated in the field work. Y-YZ and R-PW revised the paper, and all authors have read this paper and approved the final manuscript.

## Conflict of Interest

The authors declare that the research was conducted in the absence of any commercial or financial relationships that could be construed as a potential conflict of interest.

## Publisher's Note

All claims expressed in this article are solely those of the authors and do not necessarily represent those of their affiliated organizations, or those of the publisher, the editors and the reviewers. Any product that may be evaluated in this article, or claim that may be made by its manufacturer, is not guaranteed or endorsed by the publisher.

## Abbreviations

ADL: Activity of Daily Living
CNSSS: China National Stroke Screening Survey
CT: computed tomography
ICD-10: International Classification of Diseases 10th Revision
IS: ischemic stroke
MMSE: Mini-Mental State Examination
MoCA: Montreal Cognitive Assessment
MRI: magnetic resonance imaging
mRS: modified Rankin Scale
PHQ-9: Patient Health Questionnaire-9
ESRS: Essen Stroke Risk Score.
